# Management of Transvenous Leads in Patients With Iatrogenic Lead Perforation

**DOI:** 10.1111/jce.70006

**Published:** 2025-07-20

**Authors:** Arwa Younis, Ameer Awashra, Maria Matteo, Ayman A. Hussein, Joe Demian, Pasquale Santangeli, Thomas Callahan, David O. Martin, Mohamad Mdaihly, Shady Nakhla, Mohamed Kanj, Arshneel Kochar, Shinya Unai, Michael Z. Tong, Jakub Sroubek, Walid I. Saliba, Bryan Baranowski, Mina K. Chung, Oussama M. Wazni, Tyler Taigen

**Affiliations:** ^1^ Cardiac Electrophysiology Section, Department of Cardiovascular Medicine Cleveland Clinic Cleveland Ohio USA; ^2^ Department of Thoracic and Cardiovascular Surgery Cleveland Clinic Cleveland Ohio USA

**Keywords:** cardiac implantable electronic device, electrophysiology lab, iatrogenic complications, lead perforation, percutaneous lead removal, pericardial effusion, transvenous lead extraction

## Abstract

**Introduction:**

Iatrogenic lead perforation is a rare but serious complication of cardiac implantable electronic device (CIED) implantation. Evidence on percutaneous management of subacute or delayed cases remains limited.

**Methods:**

We retrospectively reviewed 38 patients treated for iatrogenic lead perforation between January 2012 and October 2024. Acute lead perforation cases, which were managed during the implantation procedure, were excluded. Lead removal was performed under imaging guidance in either the electrophysiology (EP) lab or a hybrid operating room (OR) with surgical backup. Clinical presentation, imaging, procedural approach, and outcomes were analyzed.

**Results:**

Of 38 patients, 25 underwent lead removal in the EP lab and 13 in the OR. The mean age was 67 ± 15 years; 58% were female. Most perforated leads were pacemaker leads (58%), typically involving the right ventricle (72%). Complete removal and reimplantation were achieved in all patients using simple traction with or without a locking stylet. Pericardiocentesis was required in 8% of cases. No major complications or delayed events were observed over a median 16‐month follow‐up.

**Conclusion:**

Percutaneous removal of perforated leads is feasible and safe in the EP lab when performed by experienced operators. Surgical backup should be readily available given the potential consequences of this complication.

1

Cardiac perforation represents one of the most serious complications associated with cardiac implantable electronic devices (CIED) implantations. Incidence rates are reported to range from 0.1% to 0.8% for pacemaker leads and from 0.6% to 5.2% for defibrillator leads [1]. Various management strategies for this complication have been proposed in the literature; however, due to the small number of these cases, there is no definite consensus regarding management.

This retrospective, single‐center cohort study aimed to assess the feasibility, safety, and clinical outcomes of percutaneous management of subacute and delayed transvenous lead removal in patients with iatrogenic cardiac perforation. Conducted at the Cleveland Clinic Main Campus, this analysis included all patients treated for cardiac perforation between January 2012 and October 2024, with procedures performed in either the Electrophysiology (EP) lab or a hybrid operating room (OR) with cardiac surgical backup. Patients were grouped based on the location where the lead removal procedure was performed. Cases of acute lead perforation, occurring and managed during the implantation procedure, were excluded. The Cleveland Clinic Institutional Review Board approved this retrospective medical records review study.

Cardiac perforation was suspected in patients presenting with symptoms, such as chest pain, dyspnea, extracardiac muscle stimulation, pericardial effusion, altered electrical parameters on device interrogation, or imaging evidence of lead migration. All patients initially underwent chest X‐ray and echocardiography with computed tomography (CT) used selectively in some patients for confirmation. Perforation was confirmed by visualizing the lead tip outside cardiac boundaries on imaging (Central illustration 1). To ensure no pericardial effusion had developed or worsened, echocardiography was repeated at the end of each procedure and again within 24 h postprocedure at least once.

**Central illustration 1:**
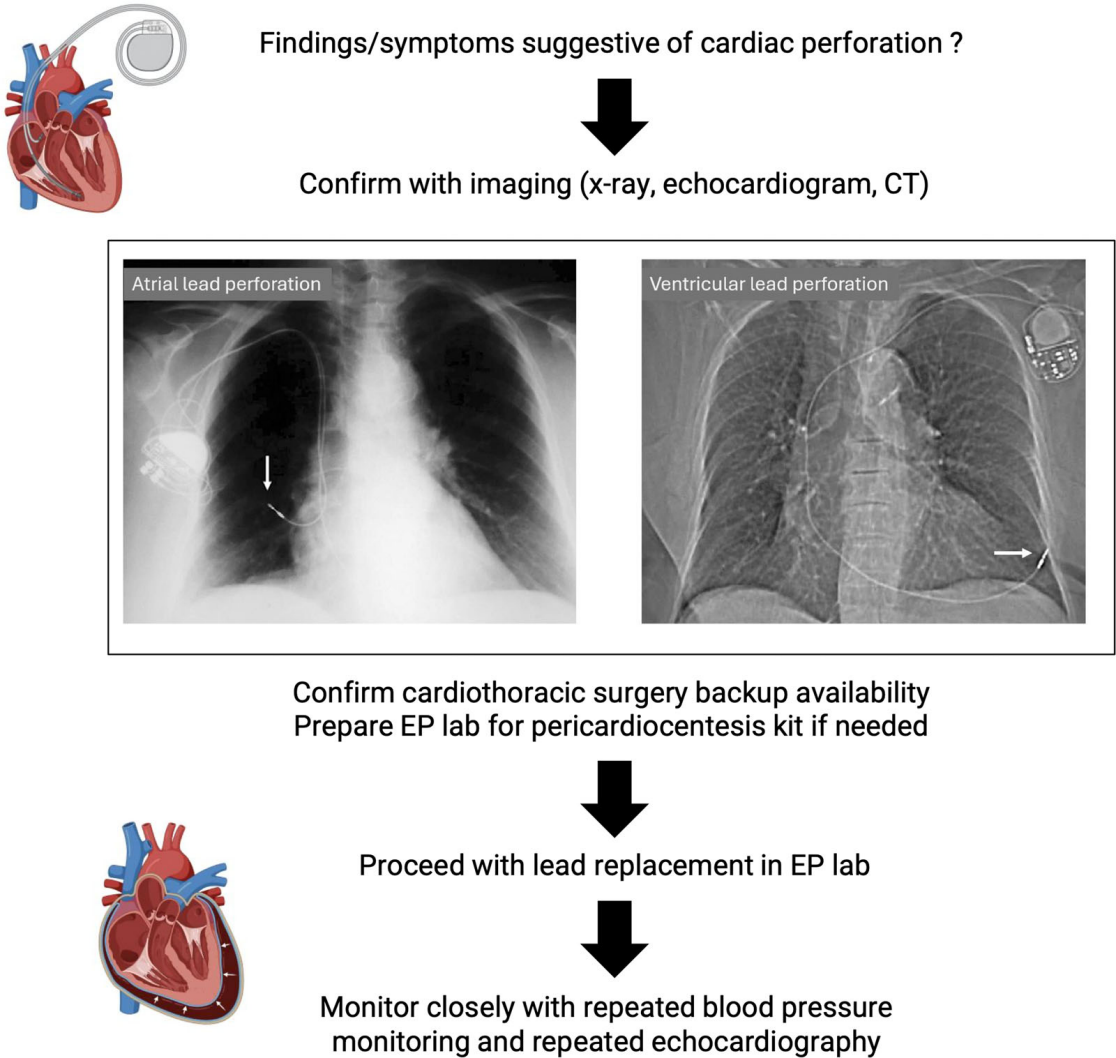
Percutaneous Management of Transvenous Lead Perforation. Central illustration demonstrating the approach to suspected iatrogenic lead perforation. Diagnosis is confirmed with imaging (chest X‐ray, echocardiography, or CT), followed by preparation of the electrophysiology (EP) lab with available pericardiocentesis equipment and confirmation of cardiothoracic surgical backup. Lead removal and reimplantation are performed in the EP lab. Postprocedural monitoring includes repeated blood pressure assessment and echocardiography to detect pericardial effusion or tamponade.

The study included a total of 38 patients, with 25 managed in the EP lab and 13 in the OR. Baseline characteristics were comparable between groups; the mean patient age was 67 ± 15 years, and 58% were female. Most perforated leads were pacemaker (58%), with the most common perforation site being the right ventricle (72%). The average duration from implantation to revision was 19 ± 34 days, with a median of 5 days [IQR 1–25].

Complete removal and reimplantation of a new lead were achieved in all 38 perforated leads across both groups (EP room and OR) without any major complications. Unplanned pericardiocentesis due to clinical instability/symptoms was required pre‐procedurally in nearly 8% of the patients (two from the EP lab group and one from the OR group). While five patients had it planned during the procedure (three in the EP lab and two in OR) due to existing effusion, and one patient had it done on the following day (EP) due to new effusion. Simple traction with or without a locking stylet was sufficient for complete lead removal in all patients. During a median follow‐up period of 16 months, there were no delayed complications reported, including no recurrence of symptoms related to lead perforation or infections associated with cardiac implantable electronic devices.

In this series of patients, lead perforations were effectively managed using a percutaneous approach in both the EP lab and OR, demonstrating that lead removal can be safely performed in the EP lab without any major complications or need for urgent surgical intervention. Once cardiac perforation is recognized, lead removal and implantation of a new lead is recommended. Removal of a perforated lead can be associated with the development of acute, life‐threatening pericardial effusion. As a result, an initial open surgical approach is often recommended, which was also the preferred method in a consensus statement endorsed by the American Heart Association. However, several recent small case series studies have proposed percutaneous lead revision with surgical backup as an alternative to open surgery, although the evidence is limited [2, 3]. The results of our study are consistent with previous research, showing that all patients were successfully treated with percutaneous lead repositioning or replacement as the initial approach, without major periprocedural complications, and none required cardiothoracic intervention [2, 3]. This can be explained by the myocardial self‐sealing properties at the perforation site. Importantly, careful monitoring throughout the procedure is essential for early identification of cardiac tamponade and the potential need for urgent pericardiocentesis. The use of transesophageal or intracardiac echocardiography could further improve procedural safety.

This study is limited by its retrospective nature, the absence of a standardized imaging protocol, and the lack of data on interobserver variability, all of which may affect the diagnostic consistency in detecting perforation. Moreover, the choice to perform the procedure in the EP lab was based on the operator discretion, which introduces the potential for selection bias, as patients with less critical presentations may have been preferentially selected for percutaneous management outside the OR setting.

In conclusion, our study, with acute/subacute lead perforations, supports prior case series studies showing that perforated leads can be safely removed in the EP lab without the need for surgery, avoiding delays in care. Although no new pericardial effusions/worsening of effusion were seen intraoperatively, the consequences of this complication can be lethal. Therefore, the procedure should only be performed by operators experienced and comfortable with the potential need for urgent pericardiocentesis and in the setting of onsite readily available surgical backup. However, given the small sample size and the absence of a control group, these findings should be interpreted with caution, and larger prospective studies are still needed to validate our results.

## Conflicts of Interest

Dr. Santangeli, Dr. Wazni, Dr. Saliba, Dr. Hussein, and Dr. Callahan have received research grants and/or consultancy fees from Medtronic, Abbot, and Boston Scientific. The other authors declare no conflicts of interest.

## Data Availability

The data that support the findings of this study are available from the corresponding author upon reasonable request.
